# Right Atrial Metastatic Melanoma with Unknown Primaries

**DOI:** 10.1155/2015/483520

**Published:** 2015-02-23

**Authors:** Robin Kuriakose, Rakhi Melvani, Venkataramanan Gangadharan, Michael Cowley

**Affiliations:** Virginia Commonwealth University, 1250 East Marshall Street, Richmond, VA 23298, USA

## Abstract

A 54-year-old male with history of anemia and rheumatoid arthritis presented with a three-month history of dyspnea on exertion and lower extremity edema. Patient was referred for a transthoracic echocardiogram that revealed a large right atrial mass with reduced ejection fraction of 40% and an incidental large liver mass. Subsequent cardiac MRI revealed a lobulated right atrial mass measuring 5.4 cm × 5.3 cm with inferior vena cava compression and adjacent multiple large liver lesions confirmed to be malignant melanoma through biopsy. Interestingly, no primaries were found in the patient. PET/CT imaging displayed hypermetabolic masses within the right atrium and liver that likely represent metastases, as well as bilateral pleural effusions, most likely due to heart failure. Preoperative coronary angiogram demonstrated perfusion to the mass by a dense network of neovasculature arising from the mid right coronary artery. The cardiac melanoma was surgically removed, and the right atrium was reconstructed with a pericardial patch. After surgery, all cardiac chambers appeared normal in size and function with associated moderate tricuspid regurgitation. The patient is currently being administered ipilimumab for systemic therapy of metastatic melanoma.

## 1. Introduction

Among all possible tumors of the heart, cardiac metastases most frequently involve melanoma. Though melanoma is the most frequent cause, cardiac metastases are still considered a rare event. In a series of 70 autopsies of patients suffering from metastatic melanoma, the frequency of metastasis to the heart was 50–71% [1]. However, cardiac metastases were diagnosed in less than 1% of cases due to asymptomatic presentation in the majority of these patients [1]. Patients where a cardiac metastasis is discovered antemortem often present foremost with symptoms of metastasis in other areas, most commonly gastrointestinal [2, 3]. When cardiac symptoms are significant, surgical intervention can be utilized to improve cardiac function and prevent further life-threatening complications such as heart failure, arrhythmias, or tumor embolization from occurring. Occasionally, melanoma metastases can present without a primary lesion. In a study of melanomas of unknown primaries (MUP), 65 of 2485 (2.6%) patients diagnosed with melanoma were lacking a primary lesion [4]. With our patient, we present a rare case of cardiac and liver melanoma metastases with an unknown primary tumor.

## 2. Case Report

**Figure 1:**
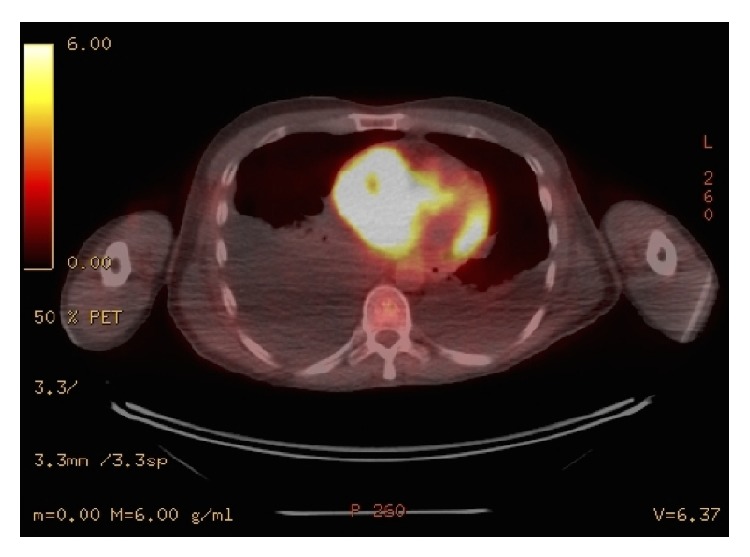
PET/CT scan displaying metastatic masses in right atrium and liver, as well as bilateral pleural effusion due to heart failure.

**Figure 2:**
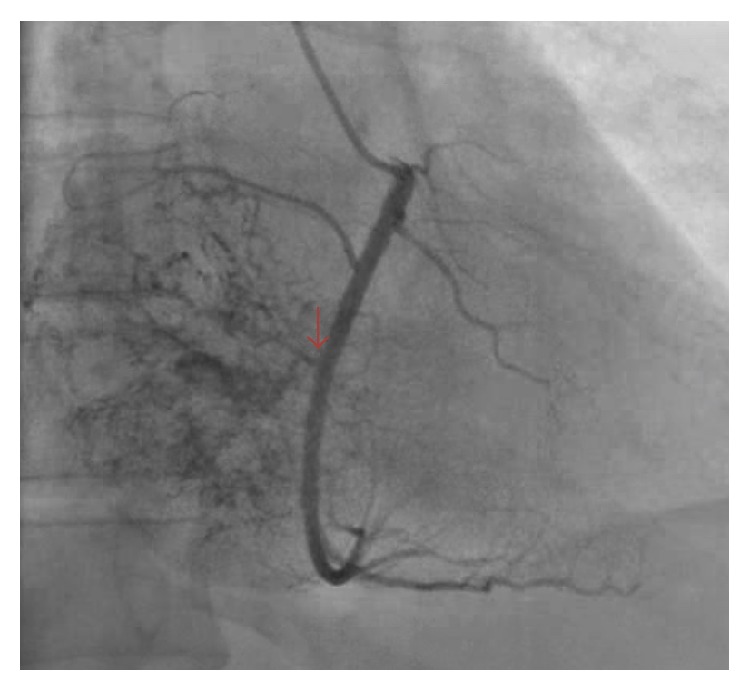
Angiogram demonstrating perfusion to the mass by a dense network of neovasculature arising from the mid right coronary artery.

**Figure 3:**
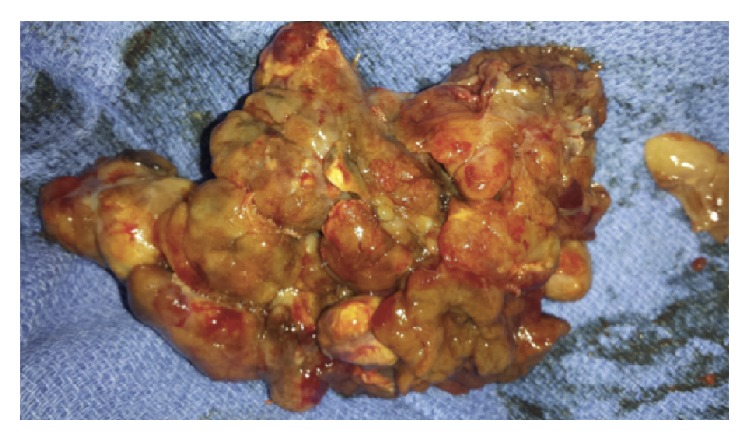
Macroscopic view of the cardiac metastatic melanoma surgically removed from the right atrium.

A 54-year-old male with history of anemia and rheumatoid arthritis had noted increased lower extremity edema, chronic cough, and shortness of breath for 3 months, at which point his rheumatologist treated him for pneumonia. With symptoms worsening, the patient visited a primary care physician, as his father had a history of coronary artery disease, colon cancer, and multiple basal cell skin cancers. The patient was then referred for a transthoracic echocardiogram (TTE), which revealed a large right atrial mass with reduced ejection fraction of 40% along with incidental lesions in the liver. A cardiac MRI and MRI of the abdomen/pelvis confirmed these results, revealing a lobulated right atrial mass measuring 5.4 cm × 5.3 cm with inferior vena cava (IVC) compression and adjacent multiple large liver lesions, the largest of which measured 6.6 cm × 7.0 cm × 7.3 cm. The cardiac mass appeared to extend through the right atrial wall and into the pericardium. The patient's abdominal MRI showed mass effect on the bile duct with central biliary duct dilatation as well as mass effect on the hepatic portal veins. Mass effect was also seen on the first portion of the duodenum causing gastric distention. After completion of both MRIs, subsequent ultrasound-guided liver biopsy returned the results of melanoma. Further workup revealed no cutaneous melanoma lesions. An eye examination was performed in order to exclude ocular melanoma. Based on the recent diagnosis of melanoma, the patient was scheduled to meet with his oncologist 4 weeks later in order to discuss treatment options. Three days prior to the patient's scheduled visit with his oncologist, the patient presented to the emergency department (ED) with worsening dyspnea and lower extremity edema. PET/CT imaging using F-18 fluorodeoxyglucose (FDG) was conducted for tumor anatomical localization. The scans displayed hypermetabolic masses within the right atrium and liver that are consistent with metastatic disease, as well as bilateral pleural effusions, likely secondary to resultant heart failure (Figure 1). Preoperative coronary angiogram demonstrated perfusion to the mass by a dense network of neovasculature arising from the mid right coronary artery (Figure 2). The cardiac mass was surgically removed, and the right atrium was reconstructed with a pericardial patch (Figure 3). Pathologic analysis of the mass confirmed melanoma. After surgery, all cardiac chambers appeared normal in size and function with associated moderate tricuspid regurgitation. Beginning one month after surgery, the patient was treated for systemic therapy with four cycles of ipilimumab, 3 mg/kg every three weeks. He subsequently developed autoimmune nephritis and a significant nephrotic syndrome as well as anasarca but recovered with pulse steroid therapy within four weeks. He had an objective response to ipilimumab in the liver and no recurrence in the heart; however, he developed progression into the peritoneal cavity, for which he is now being treated with pembrolizumab, 3 mg/kg every three weeks.

## 3. Discussion

While the skin is the most common location of a primary lesion, malignant melanomas can also be found on the mucous membranes, upper esophagus, anus, eyes, or meninges. In the present study, no primary sites were identified; rather, metastatic melanoma was diagnosed by the presence of lesions incidentally discovered on the liver and heart. While primary tumors of the heart are rare, melanomas have a strong tendency to metastasize to the heart, more commonly to the right atrium [5]. It is typical for metastases to invade the myocardium and pericardium and, more rarely, the endocardium [6]. Cardiac metastasis is rarely the initial manifestation; as many as 1% of patients' cardiac melanoma metastases go unrecognized antemortem [5]. These melanomas can metastasize to the heart through either lymphatic, hematogenous, direct extension, or intravenous extension pathways [7].

Clinical symptoms of cardiac metastasis manifest depending on the location of the metastasis and its resulting obstruction. Congestive heart failure, dysrhythmia, heart murmurs, and pericardial effusion are some of the common resulting symptoms [6, 8]. Reduction of such symptoms and prevention of further life-threatening complications as well as potential tumor embolization are driving factors in the decision to surgically resect cardiac metastases [9]. However, surgical intervention as treatment for cardiac metastases is considered palliative rather than curative [10]. Just as clinical symptoms rely on tumor location, location of the metastases in the heart also determines the efficacy of surgical treatment. In our example, the size and location of the patient's cardiac melanoma was causing IVC compression. Due to the patient's presenting symptoms of dyspnea and lower extremity edema, surgical resection was considered a priority.

Cardiac melanomas have been reported to show response to treatment when diagnosed early and aggressively. Treatment can often be divided into two modalities—medical and surgical therapy. A number of surgical resections of cardiac melanomas have been reported as successful [6, 11]. Similar reports have shown an improvement in quality of life and reduction of symptoms for at least 1 year after resection of cardiac metastases [6, 12]. However, long-term results regarding resection of cardiac metastases have yet to be evaluated, as this requires longitudinal data that is not yet present [10].

Due to this lack of data, a conventional plan for treatment has not yet been established [6]. Surgery is considered when palliative treatment is necessary and feasible, such as when location of metastases significantly impairs organ function and clear margins are attainable, as demonstrated by our patient. Medically, often in conjunction with surgical therapy, chemotherapy is the standard form of treatment. Previously, dacarbazine was the single FDA approved treatment for metastatic melanoma. In recent years, ipilimumab and vemurafenib have been successful in Phase 3 clinical trials of metastatic melanoma and have been used in practice since their FDA approval [10]. In one study, patients receiving ipilimumab alone had a median overall survival of 10.1 months (95% CI) compared to the 6.4 months (95% CI) of those treated with gp100 alone, a well-studied cancer vaccine inducing immune response [13]. Further, the reduction in the risk of melanoma progression was 36% for ipilimumab alone compared to that of gp100 alone or gp100 plus ipilimumab [13]. Based on these positive Phase 3 trial results, the patient was started on ipilimumab to treat metastatic melanoma. As the liver lesions were considered inoperable, ipilimumab was utilized as first-line therapy for the treatment of this particularly advanced melanoma.

The case presented is unique in that liver and cardiac metastases were found with no primary lesion. In a study mentioned previously, 65 of 2485 (2.6%) patients with metastatic melanoma presented without a primary lesion, making this a rare occurrence. A hypothesis regarding the origin of an MUP describes that a primary lesion can regress incidentally. Comparable patterns of the progression of metastatic melanoma with and without known primaries provide support for this theory [14]. Regression of primary melanoma could possibly be due to a forceful immune response by the host [4]. An alternative hypothesis suggests that ectopic melanocytes may have been trapped in lymph nodes or other organs inhibiting the formation of a primary lesion. This theory arises from evidence that in certain occasions benign melanocytes have been shown to reside in lymph nodes [4]. An additional theory suggests that melanocytes or melanocyte-like cells can reside in the heart [15]. It is possible in this case that a primary melanoma could have arisen from such a population [15].

With the progression of novel systemic therapies, combined with the potential benefit of surgery, outcomes for patients with cardiac melanoma metastasis are advancing. Surgery will continue to be considered an indispensable option, as it has the possibility of providing successful palliative care.
